# Risk and Environmental Factors Associated with the Presence of Canine Parvovirus Type 2 in Diarrheic Dogs from Thessaly, Central Greece

**DOI:** 10.3390/pathogens10050590

**Published:** 2021-05-12

**Authors:** Maria Kantere, Labrini V. Athanasiou, Alexios Giannakopoulos, Vassilis Skampardonis, Marina Sofia, George Valiakos, Zoi Athanasakopoulou, Antonia Touloudi, Dimitris C. Chatzopoulos, Vassiliki Spyrou, Charalambos Billinis

**Affiliations:** 1 Faculty of Veterinary Science, University of Thessaly, 43100 Karditsa, Greece; mkantere@uth.gr (M.K.); lathan@uth.gr (L.V.A.); algiannak@uth.gr (A.G.); bskamp@uth.gr (V.S.); msofia@uth.gr (M.S.); georgevaliakos@uth.gr (G.V.); zathanas@uth.gr (Z.A.); atoul@uth.gr (A.T.); dchatzopoulos@uth.gr (D.C.C.); 2 Faculty of Public and Integrated Health, University of Thessaly, 43100 Karditsa, Greece; 3 Faculty of Animal Science, University of Thessaly, 41110 Larissa, Greece; vasilikispyrou@uth.gr

**Keywords:** Canine parvovirus type 2 (CPV-2), diarrheic dogs, polymerase chain reaction, risk factors, environmental parameters, spatial analysis

## Abstract

Canine parvovirus type 2 (CPV-2) primarily infects dogs, which are the main host reservoir, causing severe gastrointestinal disease associated with immunosuppression. The present study was conducted in Thessaly, Greece and aimed to identify risk and environmental factors associated with CPV-2 infection in diarrheic dogs. Fecal samples were collected from 116 dogs presenting diarrhea and were tested by polymerase chain reaction (PCR) for the presence of CPV-2 DNA. Supplementary data regarding clinical symptoms, individual features, management factors and medical history were also gathered for each animal during clinical evaluation. Sixty-eight diarrheic dogs were found to be positive for the virus DNA in their feces. Statistical analysis revealed that CPV-2 DNA was less likely to be detected in senior dogs, while working dogs, namely hounds and shepherds, had higher odds to be positive for the virus. Livestock density and land uses, specifically the categories of discontinuous urban fabric and of human population density, were identified as significant environmental parameters associated with CPV-2 infection by using Geographical Information System (GIS) together with the Ecological Niche Model (ENM). This is the first description of the environmental variables associated with the presence of CPV-2 DNA in dogs’ feces in Greece.

## 1. Introduction

Canine parvovirus type 2 (CPV-2) of the *Parvoviridae* family is an ubiquitous virus with worldwide distribution [1,2] and is considered as a major viral pathogen of dogs. CPV-2 is a small, non-enveloped single-stranded DNA virus with three antigenic variants, CPV-2a, CPV-2b and CPV-2c [2]. The virus replicates in rapidly dividing cells of lymphoid tissues, intestinal crypt epithelial cells, precursor cells in the bone marrow and myocardiocytes in puppies under the age of one month [3].

CPV-2 causes acute enteritis with high mortality rates in young animals and also affects adults [4]. Clinical symptoms include anorexia, depression, vomiting, profuse hemorrhagic diarrhea, abdominal pain, dehydration and pyrexia, while the more severe hematological alteration is leukopenia as a result of destruction of hematopoietic progenitor cells in the bone marrow [5]. Recent reports, based on laboratory testing of feces from dogs with clinical signs, have reported prevalence of CPV-2 infection that varied among various countries from 23.6% in Netherlands, 27.7% in Spain, 53.8% in Italy, 61.5% in France, 71.4% in Germany, 70.2% in Colombia, 75% in Nigeria, 99.24% in regions of China, 91.67% in Bulgaria and 92.98% in Albania [1,6,7,8,9,10,11]. The virus is usually transmitted via fecal-oral route following contact with contaminated feces, soil or fomites, but also via predation, scavenging of carcasses, or oronasally [12]. It is shed at high titers in feces of infected animals within 4 to 5 days from exposure and for up to 10 days after recovery [13] and can persist in the environment for a time period more than a year [14], facilitating exposure of susceptible animals to infected fomites.

The most frequently reported risk factor for developing a CPV-2-caused disease is inadequate protective immunity [15]. Several factors have been reported to be associated with virus infection, including age (young < 12 months) [16], breed (purebred) [17], clinical signs as lethargy and depression [18], seasonality [16], geographical region [16], free-ranging behavior [19], inadequate veterinary health care [19], rural area of residence [16], overcrowding unsanitary environment [3,20], insufficient anthelminthic prophylaxis [21], social and financial disadvantage of owner [22,23] and lower rates of rainfall [23,24]. Moreover, a previous study in Greece reported on risk factors related to prognosis and treatment outcome [17]. In particular, prolonged duration of hospitalization was associated with dogs’ depression, vomiting, lymphopenia or hypoalbuminemia upon admission, while the syndrome of Systemic Inflammatory Response (SIRS) was correlated with poor prognosis [17].

Spatial analysis has been implemented to compare either CPV-2 prevalence in dogs between rural and urban regions by using serological methods [16,25] or exposure οf dogs to the pathogen in regions where dog-wildlife interaction occurred [19]. A recent study from Australia reported large numbers of CPV cases in dogs of rural and remote areas [23,26] by using spatial analysis combined with molecular diagnosis. Currently in Greece, there is a lack of information on environmental parameters associated with the virus presence.

The present study aimed to assess the importance of CPV-2 as a causative agent of diarrhea in dogs, to determine potential risk factors and environmental parameters associated with the virus presence and to identify possible high-risk areas by using spatial analysis for virus surveillance and control for the first time in Greece.

## 2. Results

### 2.1. CPV-2 Confirmed Infections in Diarrheic Dogs

**Figure 1:**
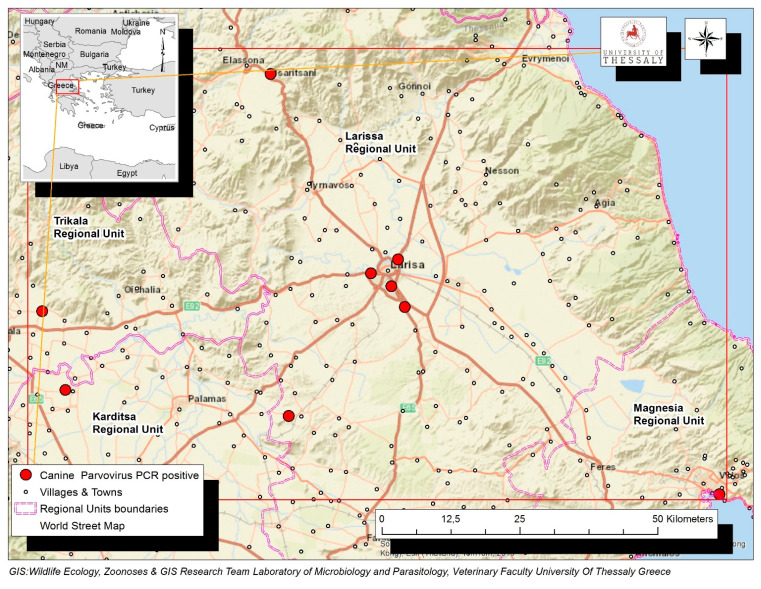
Map of Thessaly Region in Greek mainland depicting with red dots the geographical distribution of the confirmed 68 CPV-2 infections in the Regional Units of Karditsa, Larissa, Magnesia and Trikala.

A total of 116 dogs with diarrhea, originating from the Region of Thessaly in Greece, were tested by PCR for the presence of CPV-2 DNA in their feces. Virus DNA was amplified in 68 (68/116, 58.1%), while it was not detected in 48 dogs (48/116, 41.4%). Positive dogs were reported in the Regional Units of Karditsa (*n* = 19), Larissa (*n* = 15), Magnesia (*n* = 23) and Trikala (*n* = 11). The geographical distribution of the confirmed CPV-2 infections is depicted in Figure 1.

Among the 116 diarrheic dogs, CPV2-DNA was detected in 40 males (40/116, 34.5%) and 28 females (28/116, 24.1%). Furthermore, dogs were classified into three age groups and the virus presence was reported in 33 young (<1 years old, 33/116, 28.4%), 31 adults (≥1 and <9 years old, 31/116, 26.7%) and 4 seniors (≥9 years old, 4/116, 3.4%). Regarding their utility, CPV-2 DNA was identified in 16 stray animals (16/116, 13.8%), 35 companion (35/116, 30.2%) and 17 working dogs (17/116, 14.7%), namely, shepherd dogs and hounds. In respect to living environment, virus presence was confirmed in 6 dogs living indoors (6/116, 5.2%), 45 living outdoors (45/116, 38.8%) and 17 living both indoors and outdoors (17/116, 14.7%). As for close interaction with other animals, 20 positive dogs were in contact with other dogs (20/116, 17.2%), 19 with cats (19/116, 16.4%) and 29 with other animal species (29/116, 25%). At least one more clinical finding compatible with parvoviral enteritis was reported in 63 cases. In particular, 50 dogs exhibited hemorrhagic diarrhea (50/116, 43.1%), 20 vomiting (20/116, 17.2%), 18 fever (18/116, 15.5%), 12 anorexia (12/116, 10.3%), 7 hypothermia (7/116, 6%) and 4 abnormal findings in abdominal palpation (4/116, 3.4%). The results are summarized in Table 1. 

Unfortunately, information about vaccination, deworming and diet were gathered only for the categories of pets and working dogs (*n* = 85), but not for stray animals due to their unknown clinical history. Specifically, CPV-2 DNA was detected in the feces of 32 vaccinated and of 20 unvaccinated dogs, as well as in 33 dogs that received deworming therapy and in 20 that did not. In view of diet, positive dogs for the virus consumed dry food (*n* = 13), raw food (*n* = 5), homemade food (*n* = 8) or a combination of the abovementioned categories (*n* = 26).

### 2.2. Statistical Analysis

After the screening process, three parameters, namely, age, utility and presence of diarrhea, were eligible for inclusion in the full model. After model building, only two variables were significant, age and utility, and retained in the final model. Their interaction was not significant (*p* = 0.453). Hosmer–Lemeshow (*p* = 0.28) test suggested an overall adequate fit of the model to the data. Senior dogs were 4.18 (*p* = 0.034, 95% CI: 1.11–15.87) times less likely to be positive for detection of CPV-2 DNA in their feces, compared to young dogs. The odds of CPV-2 DNA detection in feces did not significantly differ between young and adult dogs (*p* = 0.246) and between adult and old dogs (*p* = 0.166). A significant association between the utility of the dog and CPV-2 DNA presence in feces was detected. Specifically, working dogs (shepherd dogs and hounds) were 13.18 (*p* = 0.015, 95% CI: 1.64–106.00) and 14.12 (*p* = 0.016, 95% CI: 1.62–122.00) times more likely to be positive for the presence of CPV-2 DNA in their feces compared to pet and stray dogs, respectively. The odds of CPV-2 DNA detection in feces did not differ between pet and stray dogs (*p* = 0.88).

### 2.3. Predictive Ecological Niche Modeling (ENM) of Canine CPV-2 Cases

**Figure 2:**
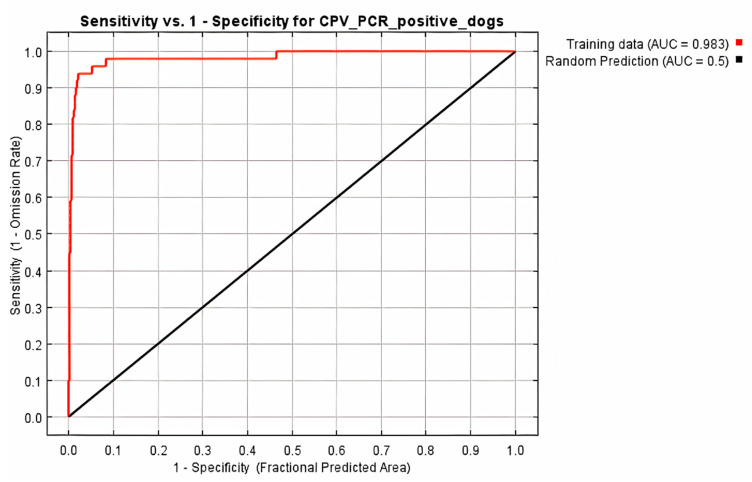
Receiver operating characteristic (ROC) curve of Maxent model for CPV-2 DNA positive dogs. The area under the curve (AUC) had value of 0.983 and exceeded that of random prediction (AUC = 0.5).

**Figure 3:**
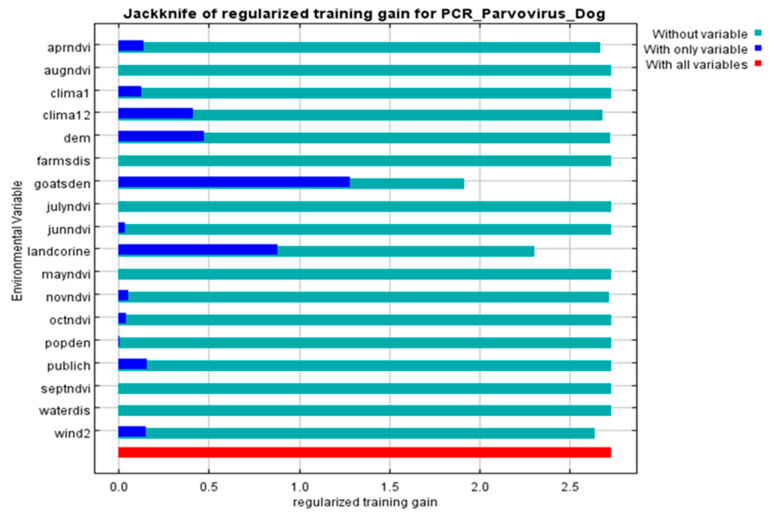
Jackknife of the regularized training gain (Maxent model) for CPV-2 DNA positive dogs. Livestock density (goatsden) and land uses (landcorine) were the environmental variables with substantial contribution to the model: without variable (light blue), with only one variable (blue), with all variables (red).

All 68 diarrheic dogs in which CPV-2 DNA was detected in their feces, were used as occurrence points for the Ecological Niche Modeling (ENM) procedure and the appropriate ecological niches for the virus presence were predicted by MaxEnt software version 3.3.3 [27]. Nine of the 34 environmental parameters included in the analysis were found to contribute to the model, as presented in Table 2. Model fitness was evaluated by a receiver operating characteristic (ROC) curve that gave a value of 0.983 for the mean area under the curve (AUC) and exceeded AUC = 0.5 of random prediction (Figure 2). Jackknife test was used to reduce the number of environmental variables to the ones with a substantial influence on the model, as it is demonstrated in Figure 3.

**Figure 4:**
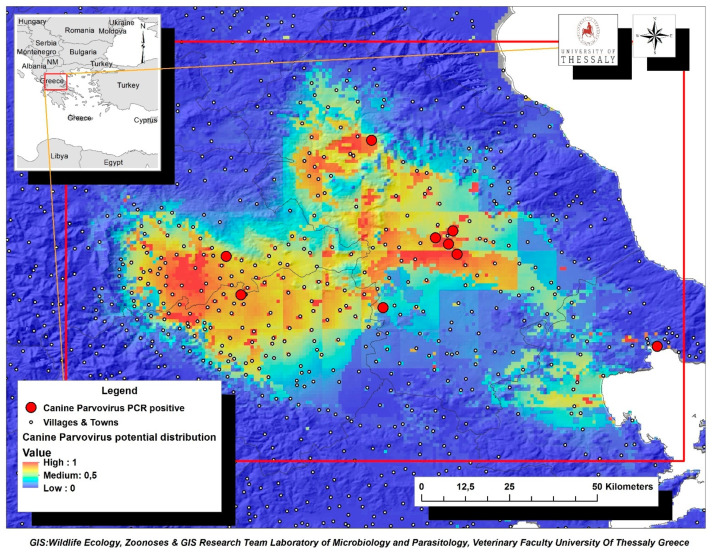
Map of Thessaly Region displaying the potential geographic distribution of CPV-2 as predicted by MaxEnt analysis based on the confirmed virus cases in dogs (red dots). Suggested high-risk areas for CPV-2 further dispersion were indicated with brown color.

The environmental variables of: (i) livestock density (goatsden), (ii) land uses (landcorin), especially the categories of discontinuous urban fabric and of agroforestry formations and (iii) human population density (popden), gave a substantial contribution to the model. Specifically, livestock density referred to the density of sheep, goat and cattle per km^–2^ and was the environmental variable with the highest gain, when it was used alone, while it decreased the gain the most, and when it was omitted. Therefore, it appeared to be the most informative variable. As for the discontinuous urban fabric class that was recognized as an additional significant environmental parameter, most of the land is covered by structures, namely buildings, roads and artificially surfaced areas, associated with the presence of vegetated areas and bare surfaces, which occupy significant surfaces in a discontinuous spatial pattern. Furthermore, the agroforestry formations class which includes land principally covered by agriculture, with significant areas of natural vegetation, was identified as an important variable. Following MaxEnt analysis that indicated the predicted probability that conditions for the presence of CPV-2 in dogs were suitable, a potential distribution of virus in dogs in Thessaly, Central Greece was depicted in Figure 4.

## 3. Discussion

The present study included 116 diarrheic dogs originating from four Regional Units of Thessaly in Central Greece, while 68 of them were found to be positive for the presence of CPV-2 DNA in their feces. Age and utility were identified as significant risk factors associated with parvoviral enteritis, whereas livestock density, land uses and human population density were described as important environmental variables related to virus infection. Favorable areas for the potential occurrence of CPV-2 infection in the region of Thessaly were also recognized. To our knowledge this is the first description of spatial analysis combined with molecular diagnosis of canine parvoviral enteritis in Greece.

Sixty-eight diarrheic dogs were considered as confirmed parvoviral enteritis cases since CPV-2 DNA was detected in their feces. The findings of the present study refer to occurrence of parvoviral disease rather than to exposure or infection by the pathogen. The compatible clinical picture and history supported potential diagnosis of parvoviral enteritis, which was confirmed by PCR. PCR is considered to be a golden standard diagnostic method [5] characterized by high specificity, although false negative results have been reported [28]. Hemorrhagic diarrhea was observed in 50 dogs, whereas at least one more compatible clinical symptom such as anorexia, fever, hypothermia and vomiting, was recorded in 63 animals. In respect of statistical analysis, the reported clinical signs as well as the findings of abdominal assessment were not associated with the virus presence in feces.

Statistical analysis also revealed that gender and living environment were not related to the presence of CPV-2 DNA in feces, as reported in previous studies [18,21]. Contacts with other dogs as well as different animal species including cats, were not associated with virus detection. Overpopulation of dogs in animal shelters has been recognized as a predisposition factor for the presence of diarrhea, not specifically caused by CPV-2 [29]. In the present study, overcrowded conditions were not reported in the cases of cohabitation with other dogs. Furthermore, dogs’ contacts with feces of cats, pigs, cattle, sheep and horses have not been related with virus occurrence [30], confirming our results for dogs living in close contact with different animal species.

Virus DNA was less likely to be detected in senior diarrheic dogs than in young ones. Several former reports have supported that dogs under the age of six months were more susceptible to develop parvoviral enteritis [3,8,9,11,15,16], although this finding was contradicted by others [31]. In view of the long-term virus persistence in the environment and its rapid intra-species transmission, the observed disease resistance in older dogs could be attributed either to their higher levels of protective immunity due to vaccination [32], a parameter that could not be evaluated in the present study, or to a lesser extent, to their infection and subsequent survival at a younger age. Nevertheless, the common empirical belief among veterinarians remains that dogs over six weeks and under six months of age are more prone to be affected by the disease [3].

A significant association of dogs’ utility and CPV-2 shedding was also detected, as the virus was more likely to be present in the feces of working dogs (hounds and shepherds) than in pet and stray ones. A previous study has identified hunting or herding activities as risk factors for CPV-2 positive results [25]. Incomplete or no vaccination has been observed more frequently in hunting and shepherd dogs, probably due to the low socio-economic background of their owners [16,23]. These dogs usually live in suburban or rural areas and tend to roam, interacting directly or indirectly with other dogs and/or wildlife species [33], which could act as potential virus sources. Furthermore, the odds of virus detection did not differ between stray and pet dogs. One could expect a higher occurrence of virus in stray dogs, as they are more often unvaccinated and live in poor conditions. Considering that they do not usually get the proper medical attention in case of illness, CPV-2 infections remain, presumably, underdiagnosed or undiagnosed, leading to severe clinical symptoms, even death. On the contrary, more pets receive treatment when and if needed, since the majority of their owners have a strong sense of responsibility to protect their health.

Spatial analysis demonstrated that livestock density was an important environmental parameter with a significant impact on the presence of canine parvoviral enteritis. Reviewing previous literature, similar results have been described by other researchers [16,34,35]. The Region of Thessaly consists of urban centers such as Larissa or Volos, as well as rural areas of crops, fields and livestock farms. Recently, the increased number of large carnivores in Greece, as in the rest of the European continent [36], led livestock breeders to keep a large number of shepherd dogs in order to minimize the damages caused by wildlife species. It has also been supported that livestock breeders tend to neglect vaccination, deworming treatment or regular visits to veterinarians probably due to the increased costs or to indifference [37]. Given that CPV-2 is a highly contagious virus that persists for a long period of time in the environment, cohabitation of unvaccinated or incompletely vaccinated dogs could lead to their infection, further contributing to the virus maintenance cycle.

Land uses, specifically discontinuous urban fabric and agroforestry, were related to the occurrence of CPV-2 infection. The former category comprises residential areas around the edge of urban district centers, and certain urban districts in rural areas, while the latter includes rural areas. A possible interpretation of virus association with suburban areas could be provided as their residents tend to be owners of guard, hunting or shepherd dogs, if they keep a few farmed animals for private use. The results of our statistical analysis further supported the aforementioned finding, since virus was more likely to be detected in hounds and shepherd dogs. A greater risk for CPV-2 infection was also reported for dogs that lived in rural areas, verifying previously published data [16,22]. Large numbers of CPV cases have been reported in rural and remote areas of Australia by using geospatial analysis [26]. In Greece, residents of rural areas are mainly farmers or livestock breeders who keep sheepdogs and have been facing financial difficulties in recent years due to economic crisis. It has been described formerly that they tend to have shorter lifespans, more difficulties making ends meet and limited access to medical facilities and education opportunities [38]. These social and economic challenges could have a negative impact on the veterinary care, diet and housing of dogs [22]. Moreover, the increased presence of unvaccinated stray or freely-roaming dogs in suburban and rural areas could contribute, in the case of their infection, to virus dissemination through interaction with healthy animals.

CPV-2 infection was also associated with human population density, as described previously by Acosta-Jamett et al. [39] who reported that dogs residing in urban areas were more likely to act as reservoirs for pathogenic infections. We believe that our finding was reasonable, since high population density usually implies an increased number of dogs, especially pets [40]. Human presence and activities could result in increased interactions between dogs, facilitating virus circulation and leading to higher infection rates [25]. Furthermore, the existence of unvaccinated stray dogs which are potential carriers of CPV-2, and live in packs and feed on uncollected garbage, leftovers or dog food [41] could contribute to virus transmission and dispersion.

This study was the first effort to simultaneously investigate possible associations of CPV-2 shedding and environmental parameters in diarrheic dogs in Greek territory. We believe that our results, as well as the map with the potential virus distribution in Thessaly Region could be used to improve the health management of both domestic and stray dogs. Interventions and educational campaigns managed by veterinarians and other stakeholders should be addressed to residents of the identified high-risk areas to promote vaccination. Considering that monitoring and understanding of CPV-2 ecology is an important tool for the determination of control and prevention measures, the results of the spatial analysis could be beneficial for dogs living in other areas with similar environmental conditions.

## 4. Materials and Methods

### 4.1. Study Area

The Region of Thessaly is divided into five Regional Units: Karditsa, Larissa, Magnesia, Sporades and Trikala, in which 25 municipalities belong, being further subdivided in 545 municipality districts. Thessaly is located in the central part of Greece and has a total area of 14,036 km^2^, which roughly represents 11% of the whole country. Thirty-six per cent of the land is flat and 17% is semi-mountainous, whereas the remaining 45% is mountainous [42].

### 4.2. Study Design

A total of 116 dogs presenting diarrhea were admitted to different veterinary clinics in four Regional Units of Thessaly (Karditsa, Larisa, Magnesia and Trikala) for clinical evaluation and treatment. Following clinical examination including detailed history, capture data forms were filled in as part of veterinary medical records held by each attending veterinarian, after receiving an informed consent from owner/routine care provider. Data included signalment (gender, age, breed and weight), factors related to animal management (dog utility, living environment, contact with animals and diet) and past medical history (vaccinations, deworming, recent disease). Recordings on diet and past medical history were not available in the cases of strayed dogs, thusly not evaluated in further analysis.

To assess the presence of possible CPV-2 infection, fecal samples were collected from each dog using a sterile cotton tipped swab suitable for virus collection and transportation (Sigma—VCM). Samples were sent frozen within 24 h for laboratory examination by PCR (Laboratory of Microbiology and Parasitology University of Thessaly, Karditsa, Greece).

### 4.3. Viral DNA Extraction

To extract viral DNA, fecal swabs were immersed in phosphate buffered saline (PBS) and centrifuged at high speed. A proportion of 200 μL from the supernatant of each sample was collected, incubated at 65 °C for 10 min to inactivate PCR inhibitors and then chilled on ice [43]. A commercial DNA purification kit (Thermo Scientific Genomic DNA Purification Kit, Waltham, MA, USA) was used to complete the extraction procedure according to the manufacturer’s protocol.

### 4.4. Molecular Detection of CPV-2

Conventional PCRs were performed using the primer pair Hfor/Hrev (Hfor: 5′-CAGGTGATGAATTGCTACA-3′, Hrev: 5′-CATTTGGATAAACTGGTGGT-3′) that amplifies a 630 bp fragment of the capsid protein-encoding VP2 gene according to Decaro et al. [44]. Each reaction mixture was adjusted in a final volume of 50 μL and contained 1× PCR buffer, 2 mM MgCl_2_, 2.5 mM of each deoxynucleotide, 0.5 μM of each primer, 2U Taq DNA Polymerase (Thermo Scientific Maxima Hot Start Taq DNA polymerase) and 5 μL of DNA extract. Cycling conditions included an initial step at 94 °C for 10 min, followed by 40 cycles of denaturation at 94 °C for 30 s, annealing at 50 °C for 1 min and extension at 72 °C for 1 min, and a final extension step at 72 °C for 10 min. Eight μL of each amplified product were analyzed by electrophoresis in a 2% agarose gel and product sizes were determined using a 100 bp DNA marker.

### 4.5. Statistical Analysis

Statistical analyses were performed using Stata 13.1 (Stata Statistical Software, College Station, TX, USA) and evaluated for significance at the 5% level. Descriptive statistics of collected data were performed. The evaluation of the association between detection (presence or absence) of CPV-2 DNA in feces with the parameters of dogs’ history, collected upon submission to veterinary clinics, was performed with the use of a logistic regression model. Presence or absence of CPV-2 DNA in feces was the dependent variable, while the parameters: (i) gender: male or female, (ii) age: young (<1 years old), adult (≥1 and <9 years old) or senior (≥9 years old), (iii) utility: companion, working or stray dog, (iv) living environment: indoors, outdoors or both, (v) contact with dogs, cats or other animal species, (vi) abdominal palpation: normal or abnormal findings, (vii) anorexia: presence or absence, (viii) hemorrhagic diarrhea: presence or absence, (ix) temperature: within reference interval, fever or hypothermia and (x) vomiting: presence or absence, were the independent covariates. All independent variables were initially screened one by one in univariate logistic regression models. During this process, a significance level of 0.25 was used as the screening criterion, since a more traditional level (such as *p* < 0.05) often fails to identify variables known to be important [45]. Then, variables with *p* < 0.25 were offered simultaneously to a full model, subsequently reduced by backwards elimination [46] until only significant (*p* < 0.05) variables remained. Two-factor interactions were created between the remaining variables and offered one at a time to the model. Finally, we offered previously deleted variables one-by-one to the final model, in order to ensure that no variable which significantly added to the model was omitted. The goodness of fit of the employed model was evaluated with the *lfit* post-estimation command.

### 4.6. Environmental Variables

Environmental variables were divided into three major classes: climatic conditions, topography and human activities. Climate indices were derived from the WorldClim version 1.4. [47], while digital elevation model (altitude) was extracted from CGIAR-CSI GeoPortal (http://srtm.csi.cgiar.org/Index.asp, accessed on 25 March 2021). Hydrological data were extracted from HydroSHEDS (https://hydrosheds.cr.usgs.gov/, accessed on 25 March 2021) and wind speed was downloaded and formatted from the Hellenic Regulatory Authority for Energy (http://www.rae.gr, accessed on 25 March 2021). Human development (municipalities/district/community) were downloaded from the Greek National Spatial Data Infrastructure (http://www.geodata.gov.gr, accessed on 25 March 2021). Land uses and human population density were derived from European Environment Agency (Copenhagen, Denmark (http://www.eea.europa.eu/data-and-maps, accessed on 25 March 2021)). The normalized difference vegetation index (NDVI) was extracted from the Copernicus European earth monitoring program (http://www.copernicus.eu, accessed on 25 March 2021) (FDC, Vincennes, France). ArcGIS 10.1 software (ESRI, Redlands, CA, USA) was used to create 34 environmental layers for the analysis. Data sets were converted to a common projection map extent and resolution prior to use in the modeling program and are presented in Table 3.

### 4.7. Ecological Niche Modeling (ENM)

Maximum entropy modeling (MaxEnt software ver. 3.3.3) [27] was used to predict the appropriate ecological niches of positive CPV-2 DNA dogs. MaxEnt method requires presence-only data, utilizes both continuous and categorical data and includes efficient deterministic algorithms and mathematical definitions [27]. CPV-2 positive dogs were used as occurrence points for the ENM procedure. The goodness of fit of the model predictions was evaluated by the mean area under the curve (AUC) of the receiver operating characteristic (ROC) curve by giving an estimation between 0 and 1 for the probability of virus presence. Jackknife test option was used to eliminate the number of environmental variables to those that exhibited a substantial influence on the model.

## Figures and Tables

**Table 1:** CPV-2 positive and negative dogs in association with ten factors and their respective categories.

Factors	Categories	Number of Dogs		
		Total	CPV-2 Positive	CPV-2 Negative
Gender	Male	66	40	26
	Female	50	28	22
Age *	<1 years old	48	33	15
	≥1 and <9 years old	54	31	23
	≥9 years old	14	4	10
Utility *	Pet	67	35	32
	Working dog	18	17	1
	Stray dog	31	16	15
Living environment	Indoors	13	6	7
	Outdoors	73	45	28
	Both	30	17	13
Contact	Dog	31	20	11
	Cat	33	19	14
	Other animal species	52	29	23
Abdominal palpation	Normal findings	105	64	41
	Abnormal findings	11	4	7
Anorexia	Presence	22	12	10
	Absence	94	56	38
Hemorrhagic diarrhea	Presence	80	50	30
	Absence	36	18	18
Body Temperature	Within reference interval	78	43	35
	Fever	27	18	9
	Hypothermia	11	7	4
Vomiting	Presence	35	20	15
	Absence	81	48	33

* Statistically significant factors are shown with background gray color.

**Table 2:** Contributions of the environmental variables to the Maxent model including percent contribution and permutation importance.

Environmental Variable	Code	Percent Contribution	Permutation Importance
Livestock density	goatsden	50.9	76.3
Human population density	popden	27.6	1.5
Land uses	landcorine	12.8	3.7
June NDVI ^1^	junendvi	2.6	3.8
Distance from small ruminant farms	farmsdis	1.8	0
Altitude	dem	1.3	0.3
Annual mean temperature	clima1	1.3	11
Total annual precipitation	clima12	0.8	2.9
Distance from water collections	waterdis	0.5	0.2

^1^ NDVI: normalized difference vegetation index.

**Table 3:** The 34 environmental variables included in GIS analysis.

Code	Environmental Variable
*clima1*	Annual mean temperature (°C)
*clima2*	Mean diurnal temperature range (°C)
*clima3*	Isothermality (*clima2*/*clima7* × 100)
*clima4*	Temperature seasonality (standard deviation × 100)
*clima5*	Maximum temperature of warmest month (°C)
*clima6*	Minimum temperature of coldest month (°C)
*clima7*	Temperature annual range (°C)
*clima8*	Mean temperature of wettest quarter (°C)
*clima9*	Mean temperature of driest quarter (°C)
*clima10*	Mean temperature of warmest quarter (°C)
*clima11*	Mean temperature of coldest quarter (°C)
*clima12*	Total annual precipitation (mm)
*clima13*	Total precipitation of wettest month (mm)
*clima14*	Total precipitation of driest month (mm)
*clima15*	Precipitation seasonality (coefficient of variation)
*clima16*	Total precipitation of wettest quarter (mm)
*clima17*	Total precipitation of driest quarter (mm)
*clima18*	Total precipitation of warmest quarter (mm)
*clima19*	Total precipitation of coldest quarter (mm)
*wind2*	Annual mean wind speed (m s^−1^)
*dem*	Altitude (m)
*waterdis*	Distance from water collections (m)
*farmsdis*	Distance from small ruminant farms (m)
*goatsden*	Sheep, goat and cattle density (animals km^−2^)
*landcorine*	Land use (principal)
*aprndvi*	April NDVI ^1^
*mayndvi*	May NDVI
*junendvi*	June NDVI
*julyndvi*	July NDVI
*augndvi*	August NDVI
*sepndvi*	September NDVI
*octndvi*	October NDVI
*novndvi*	November NDVI
*popden*	Human population density (people km^−2^)

^1^NDVI: normalized difference vegetation index.

## Data Availability

Most data are presented in this study. The remaining data are available on request from the corresponding author.
